# Assessment of patterns and related factors in using social media platforms to access health and oral health information among Sri Lankan adults, with special emphasis on promoting oral health awareness

**DOI:** 10.1186/s12889-024-19008-5

**Published:** 2024-06-01

**Authors:** Yovanthi Anurangi Jayasinghe, Kehinde Kazeem Kanmodi, Rasika Manori Jayasinghe, Ruwan Duminda Jayasinghe

**Affiliations:** 1 Department of Oral Medicine and Periodontology, Faculty of Dental Sciences, University of Peradeniya, Peradeniya, Sri Lanka; 2 School of Dentistry, University of Rwanda, Kigali, Rwanda; 3 Faculty of Dentistry, University of Puthisastra, Phnom Penh, Cambodia; 4 School of Health and Life Sciences, Teesside University, Middlesbrough, UK; 5 Cephas Health Research Initiative Inc, Ibadan, Nigeria; 6 Department of Prosthetic Dentistry, Faculty of Dental Sciences, University of Peradeniya, Peradeniya, Sri Lanka

**Keywords:** Social media, Oral health, Oral health promotion, Survey, Impact, Sri Lanka

## Abstract

**Background:**

Social media has evolved beyond its conventional purpose of communication and information sharing to become a potent tool for disseminating health and oral health awareness. This study seeks to assess the patterns and related factors of using social media platforms to access health and oral health information among Sri Lankan adults, with special emphasis to promotion of oral health awareness.

**Methods:**

In March 2023, individuals aged ≥ 18 years residing in Sri Lanka, who are users of social media participated in this electronic questionnaire-based survey. Statistical analyses of the collected data were done using the SPSS version 21 software, with a p-value of < 0.05 set to determine the level of statistical significance.

**Results:**

A total of 421 persons participated in this survey. Majority (68.4%) belonged to the age category of 18 to 30 years, and 55.5% were females. WhatsApp (96.8%) was the most frequently used social media platform across all age groups and both genders. Statistically significant differences were identified between genders in the usage of Telegram, Twitter, and Viber within the 18–30 years age category, with a higher percentage of males using these platforms (*p* ≤ 0.05). Similar significant differences were observed in the 31–40 years age group for WhatsApp and Telegram (*p* ≤ 0.05). Among 95.4% of online health information seekers, YouTube (74.9%) was the most popular platform. One-quarter of the respondents preferred social media platforms, and 22.3% preferred websites for obtaining oral health information. Furthermore, 74.9% had positive opinions on obtaining oral health information via social media, while only 17% reported pleasant experiences with social media platforms for oral health promotion. In assessing the reliability of oral health information on social media, 48% relied on the quality of the information. The most preferred source of oral health information was short videos from professionals (43.1%). Additionally, 69.5% reported changes in their oral health behaviours after accessing information through social media.

**Conclusion:**

Social media is a viable platform for promoting public oral health awareness in Sri Lankan; hence, workable strategies need to be employed, to further ensure its effective and wider use in a culturally and socioeconomically diverse country like Sri Lanka.

**Supplementary Information:**

The online version contains supplementary material available at 10.1186/s12889-024-19008-5.

## Introduction

The term social media refers to a set of technological tools in socialization and information sharing [1]. Within a short period, digital technology has taken the world to the next level with its developments, with social media becoming an essential element of modern life. Social media platforms have become most sought-after platforms in sharing or obtaining information in the forms of videos, images, audios, and texts [2]. There are diverse social media platforms. Presently, the most widely used and the most prominent platforms in the world, including Sri Lanka, are Facebook®, Instagram®, Imo™, Snapchat™, Telegram™, TikTok™, Twitter™ (now “X”), Viber™, WhatsApp®, and YouTube™. These platforms are helpful for the purpose of education [3], business [4], entertainment, and marketing [5]. In Sri Lanka, there were around 7.2 million (32.9%) social media users in January 2023 and a 3.8% yearly growth rate in this population was observed around 2021 to 2022 [6, 7]. However, it is pertinent to note that in Sri Lanka, due to less established infrastructures, the usage of social media is not widespread in the country unlike in neighbouring countries such as India [6].

As a result of the increasing use of social media platforms in Sri Lanka, the demands for traditional communication platforms such as television, radio, newspapers, and magazines have significantly declined [8]. Compared to traditional communication platforms, accessibility of social networking via social media platforms is ideal for reaching the public [9]. In terms of cost and efficiency in promoting services and products, the use of social media platforms in product marketing is very advantageous [10, 11]. Prior to the coronavirus disease 2019 (COVID-19) pandemic, the use of social media platforms in healthcare promotion was controversial; however, during pandemic situations, such controversies have been abated as more healthcare professionals are now engaged on social media [12, 13]. At present, public health specialists and advocates, including doctors, have adopted the use of social media platforms in disseminating health information to the general population to increase health awareness and to facilitate patients’ visits to the clinic for appointments and care [14]. Apart from that, social media can be utilized for advocating for health policy. Thereby active involvement with advocates is a crucial component of health policy advocacy, and social media serves as a platform for attracting new supporters and raising awareness among the general public about important health issues. An effective use of social media as an advocacy tool necessitates critical awareness, the establishment of relationships, and active engagement in mobilizing actions [15]. To achieve success in social media campaigns for health policy advocacy, public health education specialists must employ planning and evaluation skills to thoroughly assess the effectiveness of social media utilization. In addition, to engage diverse audiences effectively, healthcare professionals must adopt strategies such as comprehending the social media usage patterns of the target population, recognizing evidence-based social media strategies, selecting suitable communication times and channels, and discovering the most effectively engaged social media apps by the target audience [16].

Oral health problems are considered as a global public health burden due to their high prevalence and negative impact on individuals and society. Despite the high preventability of oral health problems and the availability of effective treatments, oral health problems have increased across different populations around the world [17]. Recently, the World Health Organization (WHO) Global Oral Health Status Report described that nearly 3.5 billion people worldwide are affected by oral health problems [18]. In the same report, it was estimated that about 29.4% of the world population had untreated caries of the permanent teeth, 9.8% had severe periodontitis, 7.8% had untreated caries of deciduous teeth and about 3.3% had complete edentulism [18]. According to the Sri Lankan Ministry of Health’s Oral Health Report 2020/2021, the prevalence of dental caries (92.5%) and calculus (70.5%) among people within the age group of 35 to 44 years is extremely high [19]. Disproportionately, oral disease burden is polarized across socioeconomic groups, and this situation is not only limited to low- and lower-middle-income countries (including Sri Lanka), but also in high-income countries [20]. To minimize the increasing burden of oral diseases, it is important to invest in low-cost public health strategies geared towards oral health promotion and oral disease prevention.

Popular social media platforms are gaining acceptance as promising approaches for oral health promotion. Several studies have been conducted worldwide on the role of social media in oral health promotion [21–26]. Additionally, several studies have been conducted in Sri Lanka on the applications of social media on disease prevention and control, and on health promotion. For example, a cross-sectional survey by Lwin et al. [27] assessed the social media-based civic engagement solutions for dengue prevention in Colombo, Sri Lanka. In another survey-based study by Lwin et al. [28], social media mobile health solution to address the needs of dengue prevention and management in Sri Lanka was assessed. A qualitative study by Vithana et al. [29] assessed adolescents’ perception of the need, acceptability, and suggestions on establishing healthy lifestyles among adolescents through e-health and m-health interventions using web-based platforms in Sri Lanka. A quantitative study by Nagahawatta et al. [30] investigated the role of social media on leaders, health-focused organisations, and community reactions across various stages of the COVID-19 pandemic in Sri Lanka. However, there is no known study that has investigated the use of social media on oral health promotion in Sri Lanka.

Oral health is essential for overall health and well-being; hence, the promotion of good oral health among adults is crucial for preventing oral diseases and maintaining quality of life. In contemporary digital age, social media has become an integral component of people’s lives, influencing their behaviours, perceptions, and choices [31]. Sri Lanka, like many other countries, has witnessed a significant increase in social media usage in recent years [7, 32]. This growth offers a unique opportunity to explore the potential of social media on oral health promotion among Sri Lankan adults. Supported by existing evidence on the prevalence of oral health problems in Sri Lanka [33–35], and given the influence of social media on health behaviours, the promotion of oral health via social media platforms may be considered as an important method, and its use seems to be a way forward [36, 37]. Apart from that, real time engagement and feedback from dental professionals on oral health information on social media platforms, bridging information gaps on oral health and formulating effective future strategies in prevention of oral diseases have been very successful [38, 39]. Therefore, it is justifiable to conduct a study to investigate the use of social media on accessing health information with special emphasis to oral health promotion among Sri Lankan adults who are the predominant users of social media in the country. Overall, this study aims to assess patterns and related factors of using social media platforms to access general health and oral health information among Sri Lankan adults, with special emphasis to the promotion of oral health awareness. The findings of this study will provide useful baseline information to frame policies and practices on wider use of social media on oral health promotion in Sri Lanka.

## Methods

### Study design

This study adopted a cross-sectional research design.

### Study population

The study participants were individuals aged 18 years or above who are residing in Sri Lanka, and who are users of free social media platforms.

### Study instrument

The study instrument was an anonymous questionnaire which was adapted from previously published literature [23, 40, 41]. Prior to its use for the main data collection, the questionnaire underwent translation, content validation, face validation, and pilot testing processes [42]. Originally, the initial questionnaire was first prepared in English language, after which a content and face validation was done for the English version. For the content validation, the initial questionnaire was reviewed by a group of 5 subject matter experts who were invited by the research team to review it. The content validation was carried out by examining if the questionnaire fulfilled the purpose of the study, clear instructions, and its relevance to the topic. Based on the feedback obtained from these experts, the questionnaire was further refined. After the content validation has been accomplished, face validation was done to assess the clarity, comprehensibility, and appropriateness of the questionnaire for the target-group. The updated version of the English questionnaire was piloted among ten participants. Based on the feedback obtained from these participants, the questionnaire’s content was further refined. As the research team aims to minimize language barriers and to maximize participation in the survey, the refined questionnaire (which was in English) was translated into Sinhala and Tamil languages by language experts. To ensure content validity, the translated questionnaires were compared with the original questionnaire (which was in English) to confirm that the intended meaning was preserved. Thereafter, the Sinhala and Tamil versions were piloted among five participants of each in Sinhala and Tamil. The finalized questionnaire had twenty-two close-ended and open-ended questions, and they were divided into four sections. The first section obtained the socio-demographic data (age, gender, province of residence, highest level of education and profession) of the participants. The second section obtained information on the usage frequency and general purposes (news, seeking or sharing information, gaming, learning, health and wellbeing, relieve boredom, business and socialization) of social media platforms. The third section obtained information on usage of social media in general health matters such as overall health, emergency treatment, medicine for common ailments, physical exercises, dietary advice, cancers, COVID-19, men’s and women’s health, juvenile health, reproductive education, and appearance. The fourth section obtained information on usage of social media in oral health matters. Emphasizing on section four, we assessed the suitable social media, searching frequency, perception, pros and cons on oral health information via social media, types of oral health information and factors considered in assessing the reliability of oral health information on social media. The reliability of oral health information relayed on social media platforms were assessed via a Likert scale of 1 to 5, where 1 referred to “strongly disagree”, 2 referred to “disagree”, 3 referred to “neutral”, 4 referred to “agree”, and 5 referred to “strongly agree”. Apart from that, preference between social media, change in oral health behaviours after accessing social media and preference mode for dissemination of oral health information were evaluated. The preference to obtain oral health information via social media were evaluated by a Likert scale of 1 to 5, where 1 referred to “strongly not preferred”, 2 referred to “not preferred”, 3 referred to “neutral”, 4 referred to “preferred”, and 5 referred to “strongly preferred” (Supplementary file).

### Sample size

The sample size of this study was calculated using Kish Leslie’s formula mentioned as follows.

$$n=\frac{{Z}_{\alpha }^{2}\text{*}\text{p} \text{*}\text{q}}{{d}^{2}}$$

According to the equation, standardized normal deviate denoted as *Z* (at 0.95 confidence level), prevalence of interest denoted as *p*, (1-*p*) indicated as *q*, clinically expected variation was denoted as *d*. In January 2022, 38.1% of Sri Lanka’s total population were active users of social media [32]. Therefore, a prevalence of 0.381 was used for the determination of the minimum sample size for this study. Based on this information, the estimated sample size was calculated as follows.

$$n=\frac{{1.96}^{2}*0.381*0.619}{{0.05}^{2}}$$

$$n=362$$

The minimum required sample size of the current study was determined as 402, considering the anticipated non-response rate as 10%.

### Data collection

The anonymous electronic questionnaire (Google form) was circulated through different social media platforms such as Facebook, WhatsApp, and Instagram. No effort was made to promote the survey via paid platforms. In addition, social media groups of professional associations/ groups were used to circulate the questionnaire to the general public. Individual social media platforms of the authors were also used. Due to the difficulty in locating the targeted study population across Sri Lanka, authors adopted the use of convenience sampling technique to recruit the participants of the study. The time of the data collection was from 29th of March 2023 to 30th of April 2023. The responses were automatically imported to a linked Google sheet and later downloaded as an excel sheet for analysis.

### Data analysis

The completeness and accuracy of the data collected from the respondents (*n* = 421) were checked, and respondents with no data on age and gender (*n* = 10) were excluded as the identification of inter-group differences according to age and gender of the respondents was an issue of interest. All qualitative data (age, province of residence, occupation, opinion on usage, pleasant and unpleasant experiences encountered) obtained from the open-ended questions were grouped into themes and coded into quantitative data. A descriptive analysis of the obtained data was done using the Statistical Package for Social Sciences (SPSS) Version 21 software. Frequencies of sample characteristics and other variables were expressed as means and percentages. Chi square test, at the level of statistical significance *p* < 0.05, was performed to assess the associations between qualitative variables.

### Ethical considerations

Ethical clearance was approved by the Ethical Review Committee, Faculty of Dental Sciences, University of Peradeniya (Ref. No: ERC/FDS/UOP/2022/26). Participation in this study was completely voluntary and anonymous. All participants were well informed about the objectives of the study prior to participation. All participants gave informed consent before participating in the study. The informed consent of the participants was obtained by them clicking to agree to the consent statement mentioned in the Google Form. Those who did not give consent to participate in the survey were automatically exited from the electronic questionnaire.

## Results

A total of 421 social media users participated in this study and among them 411 respondents provided valid responses (97.6%). More than half (55.5%) of the respondents were females while the rest (44.5%) were males. The mean (± SD) age of the respondents was 31.10 (± 10.224) years. Majority of them were within the age range of 18 to 30 years (68.4%). The highest attained education qualification among the respondents was tertiary education (81.7%), and most (66.7%) respondents were employed (Table 1).

**Table 1:** Socio-demographic characteristics of study participants (*N* = 411)

	Total *N* (%)	Gender		Age Range				
		Male *n* (%)	Female *n* (%)	18–30 *n* (%)	31–40 *n* (%)	41–50 *n* (%)	51–60 *n* (%)	60< *n* (%)
Gender Male Female	183 (44.5) 228 (55.5)	- -	- -	120(65.5) 161(70.6)	38(20.8) 33(14.5)	14(7.7) 16(7.0)	8(4.4) 11(4.8)	3(1.6) 7(3.1)
Age Range 18–30 31–40 41–50 51–60 60<	281 (68.4) 71 (17.3) 30 (7.3) 19 (4.6) 10 (2.4)	120 (42.7) 38 (53.5) 14 (46.7) 8 (42.1) 3 (30.0)	161 (57.3) 33 (46.5) 16 (53.3) 11 (57.9) 7 (70.0)					
Mean (SD)	31.10 (± 10.224) Range (18 to 71)							
Level of Education (*n* = 393) No education Till grade 5 Up to G.C.E. O/L Up to G.C.E. A/L Tertiary Education	1 (0.25) - 1 (0.25) 70 (17.8) 321 (81.7)	1 (100) - - 40 (57.1) 139 (43.3)	- - 1 (100) 30 (42.9) 182 (56.7)	1(100.0) - 1(100.0) 43(61.43) 221(68.8)	- - - 14(20.0) 54(16.8)	- - - 8(11.43) 22(6.9)	- - - 4(5.71) 15(4.7)	- - - 1(1.43) 9(2.8)
Occupation (*n* = 375) Unemployed Student Employed Retired	16 (4.3) 97 (25.9) 250 (66.6) 12 (3.2)	7 (43.75) 29 (29.9) 119 (47.6) 8 (66.7)	9 (56.25) 68 (70.1) 131 (52.4) 4 (33.3)	13(81.25) 68(70.1) 168(67.2) 7(58.4)	2(12.5) 9(9.3) 52(20.8) 3(25.0)	- 8(8.2) 18(7.2) 1(8.3)	- 9(9.3) 7(2.8) 1(8.3)	1(6.25) 3(3.1) 5(2.0) -
Province of Residence (*n* = 405) Central Province	89 (22.0)	37 (41.6)	52 (58.4)	38(42.70)	25(28.09)	14(15.73)	6(6.74)	6(6.74)
Eastern Province	17 (4.2)	4 (23.5)	13 (76.5)	17(100.0)	-	-	-	-
Northern Province	15 (3.7)	6 (40.0)	9 (60.0)	13(86.66)	1(6.67)	1(6.67)	-	-
North Central Province	8 (2.0)	3 (37.5)	5 (62.5)	7(87.5)	-	1(12.5)	-	-
Northwestern Province	35 (8.6)	21 (60.0)	14 (40.0)	26(74.3)	6(17.1)	-	3(8.6)	-
Sabaragamuwa Province	34 (8.4)	15 (44.1)	19 (55.9)	19(55.9)	10(29.4)	4(11.8)	-	1(2.9)
Southern Province	30 (7.4)	16 (53.3)	14 (46.7)	22(73.34)	4(13.33)	3(10.00)	1(3.33)	
Uva Province	21 (5.2)	10 (47.6)	11 (52.4)	19(90.48)	1(4.76)	1(4.76)	-	-
Western Province	156 (38.5)	68 (43.6)	88 (56.4)	115(73.7)	24(15.4)	5(3.2)	9(5.8)	3(1.9)

G.C.E. O/L - General Certificate of Education Ordinary Level, G.C.E. A/L - General Certificate of Education Advanced Level, SD – Standard deviation, N- Number of cases

**Fig. 1:**
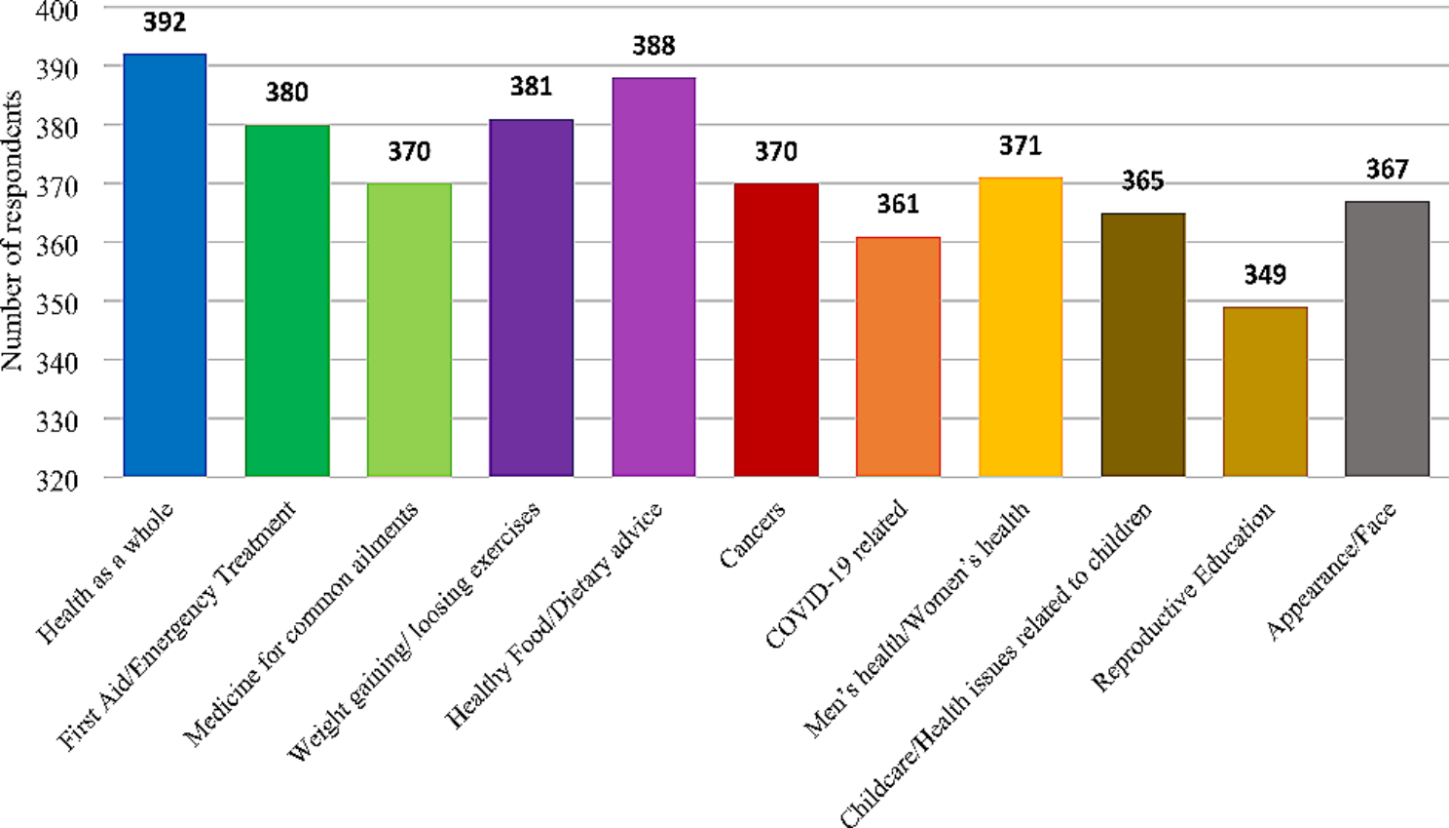
Usage of social media in obtaining information on following health matters

Facebook (73.7%), Instagram (36.7%), WhatsApp (96.7%), and YouTube (71.7%) were widely used, with high daily engagement among the respondents. Imo (60.6%), Twitter (60.3%) and other social media apps (53.8%) had lower overall usage, and majority of the respondents have never used them (Table 2). Respondents in the age category of 18 to 30 years were the most frequent users of social media (Table 3). The usage frequencies of Telegram, Twitter, Viber, and WhatsApp exhibited significant associations with gender at specific age categories. Within the Telegram and Twitter user base, significant associations were identified in the age categories of 18 to 30 years (p_Telegram_=0.037; p_Twitter_*=*0.019) and 31 to 40 years (p_Telegram_ =0.036), respectively. Among Viber users, significant associations were observed in the age category of 18 to 30 years (*p* = 0.035). Additionally, a significant association was found exclusively among WhatsApp users aged 31 to 40 years (*p* = 0.020) (Table 4). Based on the general-purpose usage of social media platforms, a notable percentage of users visited Facebook for news (*N* = 190; 46.2%), to relieve boredom (*N* = 172; 41.8%), and for conducting business (*N* = 49; 11.9%). YouTube was predominantly utilized for gaming (*N* = 64; 15.6%), learning (*N* = 171; 41.6%), and fostering health and well-being (*N* = 78; 19.0%), whereas WhatsApp emerged as the platform of choice for seeking and sharing information (*N* = 242; 58.9%), as well as for socialization (*N* = 145; 35.3%). Notably, Snapchat, Telegram, TikTok, and other social media platforms were largely utilized by females for general purposes, as per the responses (Table 5). Considering obtaining information on various health matters, majority of the respondents sought for information on overall health (95.4%), followed by information on dietary advice (94.4%), and then information on reproductive education (84.9%) (Fig. 1).

**Table 2:** Usage frequencies of social media platforms based on gender

Social Media Platforms	Total Responses *N* (%)	Missing data *N* (%)	Usage Frequencies									
			Daily *N* (%)		3–4 times a week *N* (%)		1–2 times a week *N* (%)		Rarely *N* (%)		Never *N* (%)	
			Male	Female	Male	Female	Male	Female	Male	Female	Male	Female
Facebook	372(90.5)	39(9.5)	133(48.5)	141(51.5)	11(32.4)	23(67.6)	6(42.9)	8(57.1)	10(35.7)	18(64.3)	6(27.3)	16(72.7)
Imo	249(60.6)	162(39.4)	5(100)	-	2(40.0)	3(60.0)	4(66.7)	2(33.3)	21(46.7)	24(53.3)	78(41.5)	110(58.5)
Instagram	286(69.6)	125(30.4)	46(43.8)	59(56.2)	22(52.4)	20(47.6)	14(53.8)	12(46.2)	14(38.9)	22(61.1)	32(41.6)	45(58.4)
Snapchat	253(61.6)	158(38.4)	8(44.4)	10(55.6)	8(42.1)	11(57.9)	5(38.5)	8(61.5)	17(34.7)	32(65.3)	71(46.1)	83(53.9)
Telegram	277(67.4)	134(32.6)	24(66.7)	12(33.3)	11(47.8)	12(52.2)	14(35.9)	25(64.1)	38(46.3)	44(53.7)	35(36.1)	62(63.9)
TikTok	257(62.5)	154(37.5)	21(58.3)	15(41.7)	9(39.1)	14(60.9)	5(55.6)	4(44.4)	18(54.5)	15(45.5)	64(41.0)	92(59.0)
Twitter	248(60.3)	163(39.7)	10(83.3)	2(16.7)	7(87.5)	1(12.5)	12(75.0)	4(25.0)	30(44.1)	38(55.9)	52(36.1)	92(63.9)
Viber	263(64.0)	148(36.0)	8(53.3)	7(46.7)	8(72.7)	3(27.3)	6(40.0)	9(60.0)	36(45.0)	44(55.0)	58(40.8)	84(59.2)
WhatsApp	398(96.8)	13(3.2)	170(44.2)	215(55.8)	7(70.0)	3(30.0)	1(50.0)	1(50.0)	-	1(100.0)	-	-
YouTube	389(94.6)	22(5.4)	130(46.6)	149(53.4)	31(42.5)	42(57.5)	7(29.2)	17(70.8)	5(41.7)	7(58.3)	-	1(100.0)
Other social media apps	221(53.8)	190(46.2)	11(57.9)	8(42.1)	1(20.0)	4(80.0)	4(30.8)	9(69.2)	20(37.7)	33(62.3)	61(46.6)	70(53.4)

N - Number of responses

**Table 3:** Usage frequencies of social media platforms based on age categories

Social media platforms	Total Responses N(%)	Missing data N(%)	Age Range	Usage Frequencies				
				Daily N(%)	3–4 times a week N(%)	1–2 times a week N(%)	Rarely N(%)	Never N(%)
Facebook	372(90.5)	39(9.5)	18–30	196(75.1)	17(6.5)	8(3.1)	22(8.4)	18(6.9)
			31–40	50(75.8)	8(12.1)	3(4.5)	5(7.6)	-
			41–50	18(72.0)	5(20.0)	2(8.0)	-	-
			51–60	6(42.9)	4(28.6)	-	-	4(28.6)
			> 60	4((66.7)	-	1(16.7)	1(16.7)	-
Imo	249(60.6)	162(39.4)	18–30	1(0.5)	2(1.0)	3(1.5)	36(18.3)	155(78.7)
			31–40	4(11.4)	2(5.7)	2(5.7)	7(20.0)	20(57.1)
			41–50	-	1(14.3)	1(14.3)	-	5(71.4)
			51–60	-	-	-	1(11.1)	8(88.9)
			> 60	-	-	-	1(100.0)	-
Instagram	286(69.6)	125(30.4)	18–30	91(39.6)	36(15.7)	18(7.8)	26(11.3)	59(25.7)
			31–40	10(27.0)	5(13.5)	6(16.2)	5(13.5)	11(29.7)
			41–50	2(25.0)	-	1(12.5)	3(37.5)	2(25.0)
			51–60	2(20.0)	1(10.0)	1(10.0)	1(10.0)	5(50.0)
			> 60	-	-	-	1(100.0)	-
Snapchat	253(61.6)	158(38.4)	18–30	16(7.8)	16(7.8)	11(5.4)	41(20.0)	121(59.0)
			31–40	2(6.1)	2(6.1)	2(6.1)	6(18.2)	21(63.6)
			41–50	-	-	-	1(20.0)	4(80.0)
			51–60	-	-	-	-	8(100.0
			> 60	-	1(50.0)	-	1(50.0)	-
Telegram	277(67.4)	134(32.6)	18–30	29(12.9)	20(8.9)	31(13.8)	71(31.7)	73(32.6)
			31–40	5(13.5)	3(8.1)	6(16.2)	9(24.3)	14(37.8)
			41–50	2(28.6)	-	-	2(28.6)	3(42.9)
			51–60	-	-	-	1(12.5)	7(87.5)
			> 60	-	-	-	1(100.0)	-
TikTok	257(62.5)	154(37.5)	18–30	27(13.2)	20(9.8)	7(3.4)	26(12.7)	124(60.8)
			31–40	8(21.6)	2(5.4)	2(5.4)	5(13.5)	20(54.1)
			41–50	1(16.7)	-	-	-	5(83.3)
			51–60	-	1(12.5)	-	-	7(87.5)
			> 60	-	-	-	2(100.0)	-
Twitter	248(60.3)	163(39.7)	18–30	7(3.6)	5(2.6)	14(7.1)	52(26.5)	118(60.2)
			31–40	3(9.1)	2(6.1)	1(3.0)	13(39.4)	14(42.4)
			41–50	2(22.2)	-	1(11.1)	2(22.2)	4(44.4)
			51–60	-	1(12.5)	-	-	7(87.5)
			> 60	-	-	-	1(50.0)	1(50.0)
Viber	263(64.0)	148(36.0)	18–30	6(3.0)	6(3.0)	5(2.5)	60(29.9)	124(61.7_
			31–40	8(20.0)	2(5.0)	8(20.0)	14(35.0)	8(20.0)
			41–50	1(10.0)	2(20.0)	2(20.0)	1(10.0)	4(40.0)
			51–60	-	1(10.0)	-	3(30.0)	6(60.0)
			> 60	-	-	-	2(100.0)	-
WhatsApp	398(96.8)	13(3.2)	18–30	265(97.8)	4(1.5)	1(0.4)	1(0.4)	-
			31–40	65(94.2)	4(5.8)	-	-	-
			41–50	28(93.3)	2(6.7)	-	-	-
			51–60	17(94.4)	-	1(5.6)	-	-
			> 60	10(100.0)	-	-	-	-
YouTube	389(94.6)	22(5.4)	18–30	206(75.2)	46(16.8)	12(4.4)	9(3.3)	1(0.4)
			31–40	43(67.2)	14(21.9)	7(10.9)	-	-
			41–50	13(54.2)	9(37.5)	2(8.3)	-	-
			51–60	11(57.9)	3(15.8)	2(10.5)	3(15.8)	-
			> 60	6(75.0)	1(12.5)	1(12.5)	-	-
Other social media apps	221(53.8)	190(46.2)	18–30	10(5.8)	5(2.9)	6(3.5)	41(23.8)	110(64.0)
			31–40	7(21,2)	-	6(18.2)	11(33.3)	9(27.3)
			41–50	1(16.7)	-	1(16.7)	-	4(66.7)
			51–60	-	-	-	-	8(100.0)
			> 60	1(50.0)	-	-	1(50.0)	-

**Table 4:** Association between gender and usage frequency of social media platforms in different age groups

Variable	Age categories														
	18 to 30 years			31 to 40 years			41 to 50 years			51 to 60 years			> 60 years		
	Male *N*(%)	Female *N*(%)	Statistics	Male *N*(%)	Female *N*(%)	Statistics	Male *N*(%)	Female *N*(%)	Statistics	Male *N*(%)	Female *N*(%)	Statistics	Male *N*(%)	Female *N*(%)	Statistics
Facebook	110 (42.1)	151 (57.9)	*X*^2^ = 7.132 *df* = 4 *p* = 0.129	35 (53.0)	31 (47.0)	*X*^2^ =1.526 *df* = 3 *p* = 0.676	12 (48)	13 (52)	*X*^2^ = 4.660 *df* = 2 *p* = 0.097	6 (42.9)	8 (57.1)	*X*^2^ = 0.760 *df* = 2 *p* = 0.684	3 (50)	3 (50)	*X*^2^ =2.773 *df* = 2 *p* = 0.250
Imo	83 (42.1)	114 (57.9)	*X*^2^ = 3.181 *df* = 4 *p* = 0.528	18 (51.4)	17 (48.6)	*X*^2^ = 6.845 *df* = 4 *p* = 0.144	3 (42.9)	4 (57.1)	*X*^2^ = 2.831 *df* = 2 *p* = 0.243	5 (55.6)	4 (44.4)	*X*^2^ = 1.275 *df* = 1 *p* = 1.000	1 (100)	0	-
Instagram	99 (43.0)	131 (57.0)	*X*^2^ = 3.640 *df* = 4 *p* = 0.457	19 (51.4)	18 (48.6)	*X*^2^ = 2.165 *df* = 5 *p* = 0.826	4 (50)	4 (50)	*X*^2^ = 4.499 *df* = 3 *p* = 0.212	5 (50)	5 (50)	*X*^2^ = 4.360 *df* = 4 *p* = 0.359	1 (100)	0	-
Snapchat	86 (42.0)	119 (58.0)	*X*^2^ = 3.073 *df* = 4 *p* = 0.546	16 (48.5)	17 (51.5)	*X*^2^ = 5.563 *df* = 4 *p* = 0.234	2 (40)	3 (60)	*X*^2^ = 1.185 *df* = 1 *p* = 1.000	4 (50)	4 (50)	-	1 (50)	1 (50)	*X*^2^ = 2.773 *df* = 1 *p* = 1.000
Telegram	97 (43.3)	127 (56.7)	*X*^2^ = 10.231 *df* = 4 *p* = 0.037*	17 (45.9)	20 (54.1)	*X*^2^ = 10.256 *df* = 4 *p* = 0.036*	3 (42.9)	4 (57.1)	*X*^2^ = 5.742 *df* = 2 *p* = 0.057	4 (50)	4 (50)	*X*^2^ = 1.530 *df* = 1 *p* = 1.000	1 (100)	0	-
TikTok	89 (43.6)	115 (56.4)	*X*^2^ = 2.716 *df* = 4 *p* = 0.606	20 (54.1)	17 (45.9)	*X*^2^ = 7.993 *df* = 4 *p* = 0.092	3 (50)	3 (50)	*X*^2^ = 1.588 *df* = 1 *p* = 1.000	4 (50)	4 (50)	*X*^2^ = 1.530 *df* = 1 *p* = 1.000	1 (50)	1 (50)	-
Twitter	84 (42.9)	112 (57.1)	*X*^2^ = 11.807 *df* = 4 *p* = 0.019*	17 (51.5)	16 (48.5)	*X*^2^ = 8.626 *df* = 4 *p* = 0.071	5 (55.6)	4 (44.4)	*X*^2^ = 5.094 *df* = 3 *p* = 0.165	4 (50)	4 (50)	*X*^2^ = 1.530 *df* = 1 *p* = 1.000	1 (50)	1 (50)	*X*^2^ = 2.773 *df* = 1 *p* = 1.000
Viber	85 (42.3)	116 (57.7)	*X*^2^ = 10.318 *df* = 4 *p* = 0.035*	20 (50)	20 (50)	*X*^2^ = 4.437 *df* = 4 *p* = 0.350	5 (50)	5 (50)	*X*^2^ = 6.592 *df* = 4 *p* = 0.159	5 (50)	5 (50)	*X*^2^ = 4.667 *df* = 2 *p* = 0.097	1 (50)	1 (50)	-
WhatsApp	117 (43.2)	154 (56.8)	*X*^2^ = 2.341 *df* = 3 *p* = 0.505	36 (52.2)	33 (47.8)	*X*^2^ = 5.430 *df* = 1 *p* = 0.020*	14 (46.7)	16 (53.3)	*X*^2^ = 0.010 *df* = 1 *p* = 1.000	8 (44.4)	10 (55.6)	*X*^2^ = 1.696 *df* = 1 *p* = 0.444	3 (30)	7 (70)	-
YouTube	117 (42.7)	157 (57.3)	*X*^2^ = 3.272 *df* = 4 *p* = 0.513	34 (53.1)	30 (46.9)	*X*^2^ = 2.950 *df* = 2 *p* = 0.229	12 (50)	12 (50)	*X*^2^ = 3.583 *df* = 2 *p* = 0.167	8 (42.1)	11 (57.9)	*X*^2^ = 3.805 *df* = 3 *p* = 0.283	2 (20)	8 (80)	*X*^2^ = 3.591 *df* = 2 *p* = 0.166
Other social media	71 (41.3)	101 (58.7)	*X*^2^ = 5.779 *df* = 4 *p* = 0.216	18 (54.5)	15 (45.5)	*X*^2^ = 1.258 *df* = 3 *p* = 0.739	3 (50)	3 (50)	*X*^2^ = 2.773 *df* = 2 *p* = 0.250	4 (50)	4 (50)	-	1 (50)	1 (50)	*X*^2^ = 2.773 *df* = 1 *p* = 1.000

* Statistically significant at *p* < 0.05, N- Number of responses, *X*^2^ – Pearson’s Chi Square, *df* – Degree of freedom, p – probability

**Table 5:** General purposes of social media platforms

Social media platform	Age range	General purposes N(%)															
		News		Info seeking/ sharing		Gaming		Learning		Health and Wellbeing		Relieve boredom		Business		Socialization	
		Male	Female	Male	Female	Male	Female	Male	Female	Male	Female	Male	Female	Male	Female	Male	Female
Facebook	18–30	69(53.9)	59(46.1)	76(47.2)	85(52.8)	29(82.9)	6(17.1)	30(53.6)	26(46.4)	24(54.5)	20(45.5)	64(47.1)	72(52.9)	21(58.3)	15(41.7)	54(57.4)	40(42.6)
	31–40	26(65.0)	14(35.0)	26(55.3)	21(44.7)	14(82.4)	3(17.6)	11(57.9)	8(42.1)	9(60.0)	6(40.0)	14(53.8)	12(46.2)	8(80.0)	2(20.0)	12(54.5)	10(45.5)
	41–50	5(41.7)	7(58.3)	8(47.1)	9(52.9)	2(66.7)	1(33.3)	-	3(100.0)	1(50.0)	1(50.0)	3(50.0)	3(50.0)	1(50.0)	1(50.0)	3(50.0)	3(50.0)
	51–60	4(66.7)	2(33.3)	2(25.0)	6(75.0)	1(100.0)	-	1(33.3)	2(66.7)	-	1(100.0)	2(66.7)	1(33.3)	1(100.0)	-	2(66.7)	1(33.3)
	> 60	2(50.0)	2(50.0)	3(50.0)	3(50.0)	-	-	-	1(100.0)	-	1(100.0)	-	1(100.0)	-	-	-	1(100.0)
Imo	18–30	3(60.0)	2(40.0)	2(25.0)	6(75.0)	-	-	1(50.0)	1(50.0)	1(50.0)	1(50.0)	1(33.3)	2(66.7)	1(50.0)	1(50.0)	3(16.7)	15(83.3)
	31–40	1(100.0)	-	-	-	-	-	-	-	-	-	-	-	-	-	-	5(100.0)
	41–50	-	-	-	1(100.0)	-	-	-	-	-	-	-	-	-	-	-	2(100.0)
	51–60	-	1(100.0)	-	-	-	-	-	-	-	-	-	-	-	-	-	3(100.0)
	> 60	-	1(100.0)	-	-	-	-	-	-	-	-	-	-	-	-	-	-
Instagram	18–30	12(38.7)	19(61.3)	24(47.1)	27(52.9)	2(50.0)	2(50.0)	5(35.7)	9(64.3)	3(27.3)	8(72.7)	25(46.3)	29(53.7)	6(42.9)	8(57.1)	16(30.8)	36(69.2)
	31–40	3(42.9)	4(57.1)	1(8.3)	11(91.7)	-	1(100.0)	-	3(100.0)	-	2(100.0)	5(45.5)	6(54.5)	-	1(100.0)	2(14.3)	12(85.7)
	41–50	2(40.0)	3(60.0)	3(33.3)	6(66.7)	1(100.0)	-	-	1(100.0)	-	1(100.0)	3(33.3)	6(66.7)	-	-	1(16.7)	5(83.3)
	51–60	-	5(100.0)	2(20.0)	8(80.0)	-	-	-	2(100.0)	-	3(100.0)	2(28.6)	5(71.4)	-	1(100.0)	-	2(100.0)
	> 60	-	-	-	2(100.0)	-	1(100.0)	-	-	-	-	1(33.3)	2(66.7)	-	-	-	2(100.0)
Snapchat	18–30	-	1(100.0)	-	2(100.0)	-	-	-	-	-	-	-	2(100.0)	-	1(100.0)	-	9(100.0)
	31–40	-	-	-	1(100.0)	-	-	-	-	-	-	-	-	-	1(100.0)	-	4(100.0)
	41–50	-	-	-	1(100.0)	-	-	-	-	-	-	-	3(100.0)	-	-	-	1(100.0)
	51–60	-	-	-	2(100.0)	-	-	-	-	-	-	-	1(100.0)	-	2(100.0)	-	1(100.0)
	> 60	-	-	-	1(100.0)	-	-	-	-	-	-	-	-	-	-	-	1(100.0)
Telegram	18–30	-	2(100.0)	-	9(100.0)	-	-	-	1(100.0)	-	1(100.0)	-	1(100.0)	-	-	-	10(100.0)
	31–40	-	-	-	1(100.0)	-	-	-	2(100.0)	-	-	-	-	-	-	-	5(100.0)
	41–50	-	-	-	3(100.0)	-	-	-	1(100.0)	-	-	-	3(100.0)	-	-	-	2(100.0)
	51–60	-	1(100.0)	-	3(100.0)	-	1(100.0)	-	2(100.0)	-	-	-	1(100.0)	-	1(100.0)	-	1(100.0)
	> 60	-	-	-	-	-	-	-	-	-	-	-	1(100.0)	-	1(100.0)	-	1(100.0)
Tik Tok	18–30	-	-	-	5(100.0)	-	1(100.0)	-	2(100.0)	-	1(100.0)	-	6(100.0)	-	1(100.0)	-	7(100.0)
	31–40	-	2(100.0)	-	2(100.0)	-	1(100.0)	-	1(100.0)	-	-	-	3(100.0)	-	1(100.0)	-	3(100.0)
	41–50	-	1(100.0)	-	3(100.0)	-	-	-	1(100.0)	-	-	-	4(100.0)	-	-	-	1(100.0)
	51–60	-	-	-	2(100.0)	-	-	-	-	-	-	-	-	-	-	-	2(100.0)
	> 60	-	-	-	1(100.0)	-	-	-	-	-	-	-	2(100.0)	-	-	-	-
Twitter	18–30	18(58.1)	13(41.9)	15(55.6)	12(44.4)	1(100.0)	-	-	6(100.0)	-	1(100.0)	1(25.0)	3(75.0)	4(57.1)	3(42.9)	9(52.9)	8(47.1)
	31–40	4(66.7)	2(33.3)	6(75.0)	1(25.0)	-	-	-	2(100.0)	-	-	1(100.0)	-	1(100.0)	-	-	1(100.0)
	41–50	2(100.0)	-	1(100.0)	-	-	-	-	-	-	-	-	-	1(100.0)	-	1(100.0)	-
	51–60	-	-	1(100.0)	-	-	-	-	1(100.0)	-	-	-	-	-	-	-	-
	> 60	-	-	-	-	-	-	-	-	-	-	-	-	-	-	-	-
Viber	18–30	6(54.5)	5(45.5)	9(42.9)	12(57.1)	-	-	-	3(100.0)	-	-	2(100.0)	-	2(66.7)	1(33.3)	8(32.0)	17(68.0)
	31–40	1(100.0)	-	1(33.3)	2(66.7)	-	-	-	-	-	-	-	-	-	1(100.0)	-	6(100.0)
	41–50	-	1(100.0)	-	2(100.0)	-	-	-	1(100.0)	-	1(100.0)	-	2(100.0)	-	1(100.0)	-	3(100.0)
	51–60	1(50.0)	1(50.0)	-	2(100.0)	-	-	-	1(100.0)	-	-	-	-	-	-	1(25.0)	3 (75.0)
	> 60		1(100.0)	-	1(100.0)	-	-	-	-	-	-	-	-	1(100.0)	-	-	-
WhatsApp	18–30	35(47.3)	39(52.7)	75(44.6)	93(55.4)	5(41.7)	7(58.3)	27(51.9)	25(48.1)	6(42.9)	8(57.1)	23(42.6)	31(57.4)	14(45.2)	17(54.8)	29(31.9)	62(68.1)
	31–40	10(50.0)	10(50.0)	16(47.1)	18(52.9)	1(33.3)	2(66.7)	7(63.6)	4(36.4)	2(50.0)	2(50.0)	6(54.5)	5(45.5)	6(75.0)	2(25.0)	9(33.3)	18(66.7)
	41–50	3(33.3)	6(66.7)	10(45.5)	12(54.5)	-	-	9(100.0)	-	1(33.3)	2(66.7)	1(14.3)	6(85.7)	2(50.0)	2(50.0)	3(25.0)	9(75.0)
	51–60	1(25.0)	3(75.0)	4(33.3)	8(66.7)	-	-	2(100.0)	-	1(100.0)	-	2(66.7)	1(33.3)		1(100.0)	3(33.3)	6(66.7)
	> 60	2 (66.7)	1(33.3)	2(33.3)	4(66.7)	-	-	-	-	-	1(100.0)	1(100.0)	-		1(100.0)	2(33.3)	4(66.7)
YouTube	18–30	52(31.7)	112(68.3)	63(42.3)	86(57.7)	35(77.8)	10(22.2)	55(28.6)	137(71.4)	25(41.7)	35(58.3)	48(42.5)	65(57.5)	10(66.7)	5(33.3)	12(48.0)	13(52.0)
	31–40	20(50.0)	20(50.0)	27(100.0)	-	10(100.0)	-	21(50.0)	21(50.0)	10(100.0)	-	18(100.0)	-	3(100.0)	-	1(12.5)	7(87.5)
	41–50	6(50.0)	6(50.0)	10(100.0)	-	4(100.0)	-	7(50.0)	7(50.0)	4(100.0)	-	7(100.0)	-	-	-	1(50.0)	1(50.0)
	51–60	5(50.0)	5(50.0)	5(100.0)	-	4(100.0)	-	3(50.0)	3(50.0)	2(100.0)	-	6(100.0)	-	1(100.0)	-	1(33.3)	2(66.7)
	> 60	1(50.0)	1(50.0)	1(100.0)	-	1(100.0)	-	3(50.0)	3(50.0)	2(100.0)	-	1(100.0)	-	2(100.0)	-	-	2(100.0)
Other social media	18–30	-	1(100.0)	-	5(100.0)	-	2(100.0)	-	3(100.0)	-	2(100.0)	-	-	-	-	-	8(100.0)
	31–40	-	-	-	1(100.0)	-	-	-	-	-	-	-	-	-	-	-	5(100.0)
	41–50	-	-	-	1(100.0)	-	-	-	1(100.0)	-	-	-	3(100.0)	-	1(100.0)	-	2(100.0)
	51–60	-	-	-	1(100.0)	-	-	-	1(100.0)	-	-	-	1(100.0)	-	-	-	1(100.0)
	> 60	-	-	-	-	-	-	-	-	-	-	-	1(100.0)	-	-	-	-

N – Number of responses

**Fig. 2:**
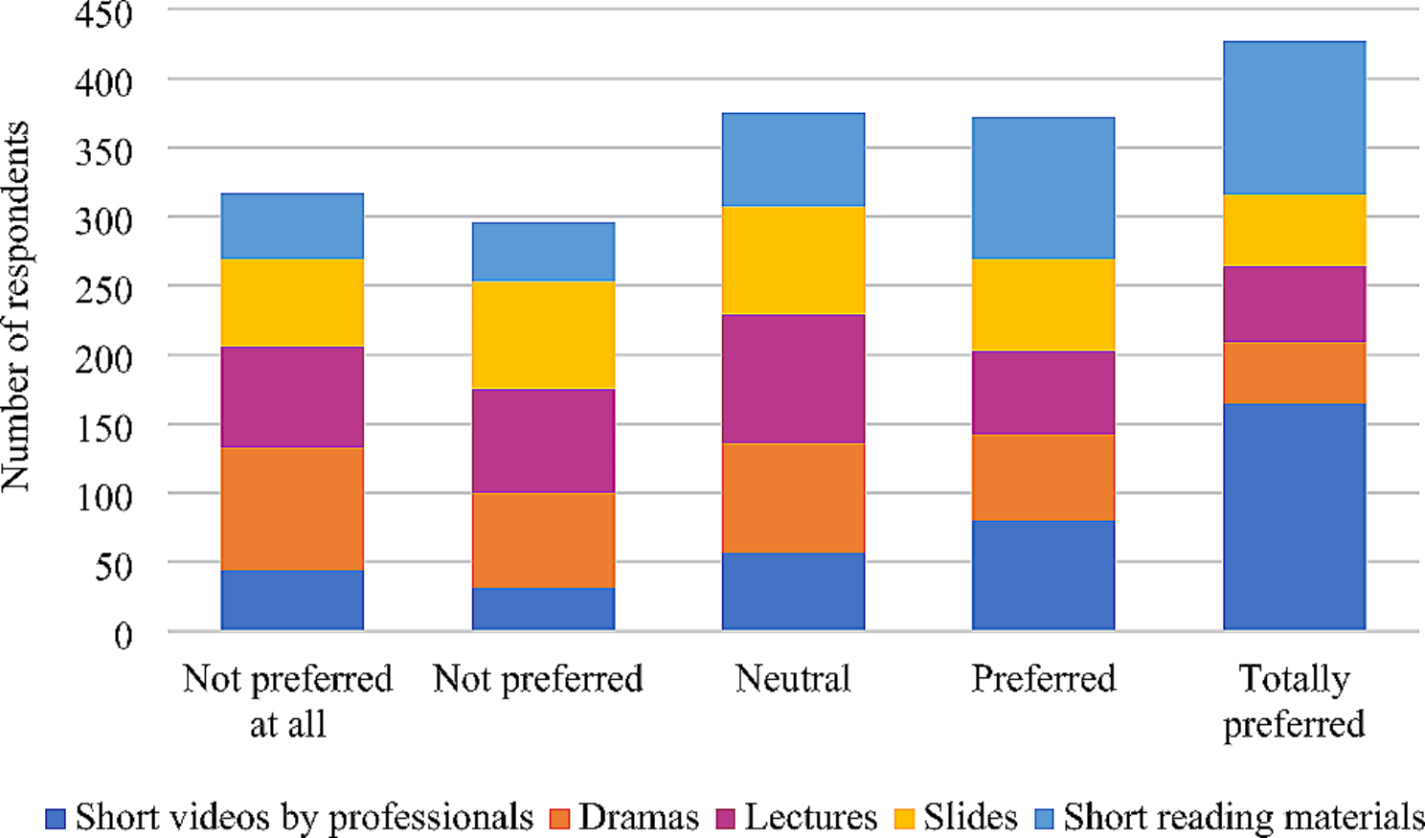
Order of preference mode for dissemination of oral health information

Regarding oral health, majority of the respondents obtained information via YouTube (74.9%) while a minute number of them obtained information via Imo (1.7%). Among the respondents, 63.4% (*n* = 258) rarely searched or viewed information on oral health. About 74.9% (*n* = 131) of the respondents had a positive opinion towards the usage of social media in obtaining oral health-related information and 63% (*n* = 189) of the respondents stated that they had no experience in using social media platforms on oral health promotion whereas only 17% (*n* = 51) noted pleasant experiences. Among the respondents, 68.7% (*n* = 279) stated accessing information on improving oral health was easy, while 66.7% (*n* = 270) stated that the accuracy of information related to oral health was questionable. Based on the types of information related to oral health, 53.2% (*n* = 199) searched on details on how to maintain oral hygiene. More than half (53.5%; *n* = 220) of the participants graded the reliability of oral health-related information found on social media as ‘neutral’ on the Likert scale of agreement and disagreement. Less than half (46.3%; *n* = 187) of the participants were satisfied with the accuracy and transparency of the information on oral health on social media, while 32.7% (*n* = 132) were concerned about the affiliation of the expert who provided such information on social media. However, 51% (*n* = 206) preferred both websites and social media for the promotion of oral health. About 69.5% (*n* = 280) stated that they have changed their oral health behaviours after accessing social media (Table 6). According to the mode of dissemination of oral health information, 43.1% (*n* = 166) totally preferred short videos provided by health professionals in the concerned subject field (Fig. 2).

**Table 6:** Frequencies among variables associated with social media platforms and oral health (*N* = 411)

	Total *N* (%)	Male *n* (%)	Female *n* (%)
Social media applications users seek to obtain information on oral health*			
Facebook	208 (50.6)	86 (41.3)	122 (58.7)
Imo	7 (1.7)	0	7 (100)
Instagram	68 (16.5)	15 (22.1)	53 (77.9)
Snapchat	9 (2.2)	2 (22.2)	7 (77.8)
Telegram	19 (4.6)	4 (21.1)	15 (78.9)
TikTok	35 (8.5)	8 (22.9)	27 (77.1)
Twitter	11 (2.7)	2 (18.2)	9 (81.8)
Viber	9 (2.2)	2 (22.2)	7 (77.8)
WhatsApp	105 (25.5)	34 (32.4)	71 (67.6)
YouTube	308 (74.9)	131 (42.5)	177 (57.5)
Other social media	58 (14.1)	25 (43.1)	33 (56.9)
Frequency of search/view oral health information			
Every few days	22 (5.4)	3 (13.6)	19 (86.4)
Every few weeks	37 (9.1)	12 (32.4)	25 (67.6)
Every few months	69 (17)	33 (47.8)	36 (52.2)
Rarely	258 (63.4)	130 (50.4)	128 (49.6)
Never	21 (5.1)	2 (9.5)	19 (90.5)
Opinion on using social media to obtain information on oral health			
Positive	131 (74.9)	54 (41.2)	77 (58.8)
Negative	29 (16.6)	12 (41.4)	17 (58.6)
Both	13 (7.4)	5 (38.5)	8 (61.5)
Neutral	2 (1.1)	1 (50)	1 (50)
Pleasant and unpleasant experiences which encountered when using social media related to oral health promotion			
Pleasant Unpleasant Both No Experience	51 (17) 41 (13.7) 19 (6.3) 189 (63)	23 (45.1) 18 (43.9) 7 (36.8) 81 (42.9)	28 (54.9) 23 (56.1) 12 (63.2) 108 (57.1)
Advantages of using social media in obtaining oral health information*			
Easy to access information	279 (68.7)	113 (40.5)	166 (59.5)
Fast and cost-effective	157 (38.7)	38 (24.2)	119 (75.8)
Applicable for all age groups	47 (11.6)	2 (4.3)	45 (95.7)
Information is sufficiently available	61 (15)	16 (26.2)	45 (73.8)
Clinics not available everywhere	31 (7.6)	2 (6.45)	29 (93.5)
Easy to share experiences with friends	73 (18)	9 (12.3)	64 (87.7)
Disadvantages of using social media in getting oral health information*			
Accuracy is questionable	270 (66.7)	114 (42.2)	156 (57.8)
Difficult to directly communicate with experts	131 (32.3)	35 (26.7)	96 (73.3)
Not enough time to use social media	21 (5.2)	3 (14.3)	18 (85.7)
No interest in oral health issues	28 (6.9)	10 (35.7)	18 (64.3)
Information available is insufficient	61 (15.1)	14 (23)	47 (77)
Other	17 (4.2)	1 (5.9)	16 (94.1)
Types of oral health information sought*			
Oral health	50 (13.4)	6 (12)	44 (88)
Oral hygiene	199 (53.2)	74 (37.2)	125 (62.8)
Oral health emergencies	65 (17.4)	20 (30.8)	45 (69.2)
Toothcare remedies	98 (26.2)	15 (15.3)	83 (84.7)
Dental caries or gum diseases	90 (24.1)	28 (31.1)	62 (68.9)
Oral health of children	44 (11.8)	17 (38.6)	27 (61.4)
Find a suitable dentist	35 (9.4)	7 (20)	28 (80)
Oral surgery	40 (10.7)	2 (5)	38 (95)
Risk habits	29 (7.8)	0	29 (100)
Reliability of oral health related information found on social media			
Totally Disagree	24 (5.8)	14 (58.3)	10 (41.7)
Disagree	68 (16.5)	36 (52.9)	32 (47.1)
Neutral	220 (53.5)	93 (42.3)	127 (57.7)
Agree	77 (18.7)	30 (39)	47 (61)
Totally Agree	21 (5.1)	9 (42.9)	12 (57.1)
Factors considered when assessing the reliability of the information on oral health on social media*			
Accuracy/ Transparency	187 (46.3)	60 (32.1)	127 (67.9)
Affiliation of person/ expertise	132 (32.7)	44 (33.3)	88 (66.7)
Quality of information	194 (48)	52 (26.8)	142 (73.2)
Number of followers	50 (12.4)	6 (12)	44 (88)
Recommendation of source by third party	68 (16.8)	10 (14.7)	58 (85.3)
Novelty/ recentness	38 (9.4)	5 (13.2)	33 (86.8)
Preference to obtain information on oral health via social media			
Not preferred at all	18 (4.4)	10 (55.6)	8 (44.4)
Not preferred	42 (10.3)	26 (61.9)	16 (38.1)
Neutral	186 (45.8)	75 (40.3)	111 (59.7)
Preferred	85 (20.9)	35 (41.2)	50 (58.8)
Totally preferred	75 (18.5)	33 (44)	42 (56)
Preference between website or social media to disseminate oral health information			
Website preferred	90 (22.3)	35 (38.9)	55 (61.1)
Social media preferred	101 (25)	55 (54.5)	46 (45.5)
Both website and social media preferred	206 (51)	84 (40.8)	122 (59.2)
None preferred	7 (1.7)	3 (42.9)	4 (57.1)
Change in oral health behaviours after accessing social media			
Yes	280 (69.5)	122 (43.6)	158 (56.4)
No	123 (30.5)	55 (44.7)	68 (55.3)

*Multiple responses

## Discussion

Several studies have been conducted in Sri Lanka on the roles of social media on health promotion; however, none of those studies were focused on oral health promotion [27–30]. Considering oral health promotion via social media, to the best of our knowledge, this was the first-ever survey of such from Sri Lanka. At present, owing to various risk factors, including poor oral health maintenance and lack of awareness, oral diseases have become a major public health burden in Sri Lanka [43]. With the rise of social media users in Sri Lanka, this research provided novel insights to bridge knowledge gaps on oral health literacy in different socio-economic groups in the country. Given the impact of economic crisis on public dental services and financial constraints in accessing private dental care services, the use of social media platforms provides a promising low-cost approach for reducing the oral disease burden and promoting oral health among Sri Lankans.

This descriptive cross-sectional survey assessed 411 respondents, and more than 50% of them were females while more than half were between 18 and 30 years of age. These findings on the age and gender distribution of the respondents in the present study were consistent with what is obtained in some similar studies conducted elsewhere [26, 44]. Further, majority of the respondents were educated, where tertiary education was the highest attained education among more than two-third of the respondents who were between 18 and 30 years of age. Similarly, more than half of the respondents of the same age group were employed (Table 1). These observations are not too surprising because, globally, the digitally savvy population are young and educated people who often lead autonomous lifestyles, striving to create lasting impact on society through their innovative approaches and technological expertise [45].

Supported by the findings of this current study and other existing evidence, WhatsApp is the most preferred social media platform for general-purpose usage (Tables 2 and 3) [26]. One of the reasons for this preference is its user-friendly interface, coupled with a wide array of messaging features (text, voice, and video communication), which appeals to those users seeking continuous and convenient communication and end-to-end encryption, ensuring privacy and security [46]. However, contrary findings on the preference and usage of WhatsApp have been reported elsewhere [23, 47]. Furthermore, majority of the respondents of the current study were from Western and Central provinces (Table 1). Nevertheless, in our study, the urban-rural division on usage of social media in health promotion was not assessed. Thus, according to the existing studies conducted elsewhere, usage of social media platforms depends only on the purpose in both urban and rural dwellers [48–50]. Thereby, it can be emphasised that the preference for social media platform differs with age, gender, country, region, and purpose; and this is due to the policy regulations of each social media platforms and online safety bills unique to each country [51].

The findings of our study highlighted the awareness, dissemination and opinion of oral health promotion compared to general health via social media platforms. According to the findings of our study, search for overall health on social media was prominent compared to the search of oral health and majority rarely viewed oral health information on social media platforms (Fig. 1; Table 6). This suggests a lack of interest and awareness among Sri Lankan population on oral health and its promotion [52, 53]. Other reasons for these extreme results would be the perception of overall health topics (fitness, nutrition and mental well-being) to be more critical to the overall well-being compared to oral health among our study cohort and whether information is sought or not, the individual anyway needs to visit a dentist for the identification of oral changes and management of them. This is further proven by the evidence in this current study where the search of nutrition and fitness information on social media were comparatively higher than the search for oral health information on social media (Fig. 1). Pertinently, regular updates on diverse oral health content, and interaction with followers on oral health promotion and awareness are essential to keep the audience engaged and interested in oral health topics to overcome the knowledge gap between overall health and oral health in Sri Lanka [38, 54].

Furthermore, majority of the respondents preferred YouTube to obtain information related to oral health and this was supported by evidence obtained from an existing study (Table 6) [26]. Utilizing captivating graphics, informative videos, and compelling infographics on social media can significantly influence oral health promotion by illustrating proper oral hygiene practices, emphasizing the importance of regular dental check-ups, and elucidating the consequences of neglecting oral health [55]. According to the findings of our study on the mode of preference for dissemination of oral health information, majority of the respondents preferred short videos made by oral health professionals (Fig. 2). However, a review of findings documented elsewhere indicated that Instagram was the most suitable social media platform in disseminating oral health information [56, 57]. The findings from those studies are contrary to our findings concerning the most suitable social media platform in disseminating oral health information, as less than one-quarter of the respondents in our study preferred Instagram to obtain oral health information. These contrary findings could be due to Instagram’s unique prominence in those countries and its ability of discovering information through hashtags by its users. As revealed by our study findings, it is important to select the suitable social media platform and the mode of dissemination of oral health information. Further, by identifying the suitable social media platform for the scope of oral health dissemination and its mode can make the general public more aware of the importance of oral health for the overall well-being of an individual.

Considering the factors considered in assessing the reliability of information on oral health in social media, the majority of the respondents in this current study relied on the quality of information, accuracy/transparency, affiliation of the experts on an oral health promotion account on social media platforms. Thus, a smaller number of respondents relied on the number of followers on the sourced social media accounts, recommendation of the source by a third party and novelty of content in oral health promotion accounts (Table 6). However, in several existing studies, majority of its respondents relied on qualification of the dentist and their social media presence when obtaining oral health information from dentists’ social media accounts [23, 47]. Thereby, it is suggested that these factors should be considered in proper regulation of online safety by establishing reputable sources. Further, the recently effected online safety bill of Sri Lanka can maintain trust between sources of social media among the social media users. Thereby, social media can be improved to obtain accurate and reliable oral health information among Sri Lankans.

This present study also assessed the most suitable information source to obtain oral health. In published studies, majority of its respondents rely on medical websites, and they preferred it over social media platforms [47, 58]. In contrast, the study respondents equally preferred both information sources, websites, and social media to obtain oral health information. Preference on websites over social media on published studies could be due to its dissemination of in-depth and credible oral health information by dental professionals, health organizations, and other reputable sources. Thus, considering these information sources separately, based on our findings, 25% of the respondents preferred social media over websites to obtain oral health information. From the findings of our study, it is already established that the respondents of our study have a positive opinion in obtaining oral health information via social media (Table 6). Following are some of the positive statements provided by the respondents in using social media platforms to obtain information about oral health: “*It is very convenient and quick method of gathering information*”; “*Can use for effective knowledge sharing*”; “*Easy access for information*”; and “*A good way to approach people*”. Apart from these positive comments few respondents stated some negative opinions regarding the reliability of oral health-related information. Examples of the negative statements stated by the respondents include “*Reliability is low*”; “*Not 100% accurate*”; and “*There are issues with the authenticity of the information*”. Thereby, the authors of this study suggest that Sri Lankan health authorities should take necessary steps to publish precise oral health information in reputable social media sources. This can be further achieved by collaboration between Ministry of Health and social media regulation authority in Sri Lanka.

Social media as a promotion platform has its own benefits. Real-time interactions, cost-effectiveness, targeted marketing, and likelihood of content spreading widely and rapidly across social media platforms are some of the benefits of utilizing social media platforms in promoting oral health [38, 59, 60]. Moreover, social media platforms offer precise targeting options, enabling oral health campaigns to reach specific demographics, including age groups, locations, and interests. This ensures that information is tailored to the needs of the target audience [61] [38, 59, 60, 62]. As a result, carefully crafted content on social media holds the capability to widely spread, raising awareness about oral health to an audience of a wide range [56]. In our study, the highly considered benefits in obtaining oral health information by social media platforms were accession of information was easy, fast and cost effective (Table 6). Based on these benefits, it can be suggested that promotion of oral health via social media platforms will be effective, with a proper social media regulation policy.

Apart from the benefits, there are some notable challenges to consider when promoting oral health via social media platforms. Majority of the respondents in our study also stated that the oral health information on social media is questionable (Table 6). This can be due to the ease of content creation on social media which can lead to the spread of inaccurate or misleading information about oral health. Further, spread of information could be affected by cultural beliefs, individual preferences, and practices [63, 64]. The public tend to circulate information if they are acknowledged within their cultural norms [65]. Further research has revealed that even oral health profession trainees consider cultural limitations when using social media to communicate with the patients [66]. Hence, it is crucial for oral health professionals and organizations to ensure that the information they share on social media is evidence-based and reliable [40, 67].

### Future recommendations

Promoting oral health presents a formidable challenge which is intersected due to Sri Lanka’s current economic challenges. Owing to the changes in the society, conventional approaches such as lectures and the use of print and electronic mass media are increasingly recognized as less effective modes. Social media has emerged as a highly impactful tool, utilized by both the general populace and healthcare professionals, proving effective platforms in both personal and professional spheres. Hence, social media can be considered as an effective tool in oral health promotion. As revealed by the findings of this study, increasing number of people are using social media as popular platform in gathering information related to oral health. Considering the fact that the respondents in this study were more interested in information on general health (compared to oral health) through social media; several key strategies are to be employed in Sri Lanka to implement effective oral health promotion on social media.

Economic challenges in Sri Lanka in the recent years have led to oral healthcare disparities across different regions in the country. Thereby, leveraging on social media platforms for policy advocacy holds immense potential in improving oral health access and awareness. The following are key strategies that can be applied by policy advocacy in establishing regularized policy frameworks concerning social media-based oral health promotion. As a priority, governmental bodies, such as the Ministry of Health Sri Lanka partnering with an established social media company can promptly help in ensuring that accurate oral health information are churned out to reach a broader audience [61]. Designing educational initiatives involves crafting themed campaigns that offer detailed insights into various oral health subjects, including dental caries and gum disease prevention, the significance of fluoride in oral health protection, proper oral hygiene techniques, the importance of routine dental examinations, and the repercussions of neglecting oral health. Additionally, sharing real-life success stories and testimonials to connect with the audience emotionally in social media platforms allow accessibility to oral health promotion [61]. Introducing games, quizzes, and challenges that encourage users to actively participate in learning about oral health have the potential to enhance the effectiveness of oral health promotion efforts; hence, these strategies should be adopted in Sri Lanka [68, 69].

The Health Promotion Bureau (HPB) of the Ministry of Health, Sri Lanka, is the focal point of health and oral health promotion in the country [19, 70]. Further, digital and social-media platforms are being increasingly utilized for health and oral health promotional activities formulated and delivered by HPB [71]. Therefore, the findings of this study will offer essential foundation for the HPB to support in the ongoing national oral health promotion efforts in Sri Lanka. To further advance oral health promotion via social media in Sri Lanka and globally, it is imperative to implement targeted strategies and initiatives. Thereby, authors of this study recommend the need to conduct research on longitudinal studies to assess the long-term effectiveness of social media interventions on oral health behaviours and outcomes among diverse populations in Sri Lanka.

### Limitations and strengths

This survey has its limitations. The sampling technique adopted in this study was based on snowballing method; a non-probability sampling technique which did not give all potential participants an equal chance to participate in the study. As a result of this, an even distribution among the provinces, rural areas and urban areas was not achieved. Another limitation was that this study may lack in-depth information about respondents’ behaviours, motivations, and experiences with oral health promotion on social media. Being an e-questionnaire (Google Form) as its survey tool, it typically allowed relatively short and straightforward responses. Additionally, in the data analysis, confounding bias was expected due to essential demographic factors such as socioeconomic status and access to healthcare services of the respondents were not assessed. It would be valuable if future studies can consider the above-mentioned demographic factors to provide further findings relevant to the Sri Lankan demographic variations. Such studies would provide further insights on how to further enhance oral health promotion through social media in Sri Lanka. Also, another limitation were the gender options as the only options on gender provided in the questionnaire were “male or female”. Among the total study cohort 10 individuals have not stated their gender and these individuals who were not identified as males or females were excluded from the data analysis. Further, social desirability bias was expected in the results as most of the data relies on the self-reporting behaviour of the individuals.

Notwithstanding the above limitations, this study has its strengths. This study adopted a descriptive cross-sectional design which is very efficient in collecting anonymous information concerning a population’s characteristics, behaviours, awareness, knowledge, and health statuses. Likewise, the current study was focused on the usage of social media and oral health behaviours in Sri Lanka, making it to be, to the best of the authors’ knowledge, the first published study of its kind in the whole of the country.

## Conclusion

The findings of this study revealed a complex and evolving landscape centred on the distribution and associations with age groups and gender on using social media in promoting oral health in Sri Lanka. Social media have demonstrated their potential as powerful tools for disseminating oral health information, fostering engagement, and reaching a wide audience at a relatively low cost. The importance of recognizing the unique socio-cultural context of Sri Lanka in establishing social media-based oral health promotion strategies were highlighted in this study. Additionally, the outcome of this study emphasizes the importance of tackling oral health inequalities, linguistic variations, and digital literacy challenges. It also highlights the need to promote a preventive dental care ethos across diverse socioeconomic strata and enhance societal awareness in the country. The findings of this study would not only be relevant to Sri Lanka only, but it will offer valuable insights into global public health initiatives seeking to utilize the potential of social media in oral health promotion. As social media continues to evolve, the importance in adapting the strategies to effectively promote oral health and address emerging challenges cannot be overlooked.

### Electronic supplementary material

Below is the link to the electronic supplementary material.

Supplementary Material 1

## Data Availability

The authors confirm that the data supporting the findings of this study is available upon request from the corresponding author.
