# Supplementary material for: Assessment of patterns and related factors in using social media platforms to access health and oral health information among Sri Lankan adults, with special emphasis on promoting oral health awareness
Source: BMC Public Health. 2024 Jun 1;24:1472. doi: 10.1186/s12889-024-19008-5 (PMC11143610; doi:10.1186/s12889-024-19008-5)
Supplement: Supplementary file 1 — Supplementary Material 1 [file 12889_2024_19008_MOESM1_ESM.docx]

**Supplementary file**

Questionnaire

**Section I** – Socio-demographic data

1. Age - …………...

| Male |  |
| --- | --- |
| Female |  |

1. Gender -
2. Province of residence - …………………………….

| No education |  |
| --- | --- |
| Up to 5th grade |  |
| Up to G. C. E. O/L |  |
| Up to G. C. E. A/L |  |
| Tertiary Education |  |

1. Highest level of education -

| Currently working |  |
| --- | --- |
| Retired |  |

1. Occupation - ………………………..
2. Status of Occupation -

**Section II** - Usage frequency and general purposes of social media platforms

1. Please mark your usage frequency of social media platforms with a (
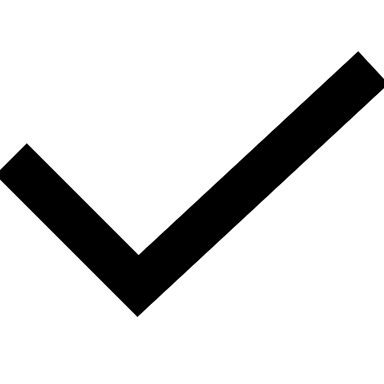
).

|  | Daily | 3-4 times a week | 1-2 times a week | Rarely | Not at all |
| --- | --- | --- | --- | --- | --- |
| Facebook |  |  |  |  |  |
| Twitter |  |  |  |  |  |
| YouTube |  |  |  |  |  |
| WhatsApp |  |  |  |  |  |
| Viber |  |  |  |  |  |
| Imo |  |  |  |  |  |
| Instagram |  |  |  |  |  |
| Tik Tok |  |  |  |  |  |
| Telegram |  |  |  |  |  |
| Snapchat |  |  |  |  |  |
| Others |  |  |  |  |  |

1. What is the purpose of using different types of social media?

|  | News | Information seeking/ sharing | Gaming | Learning | Health and well-being | Get rid of boredom | Commercial | Socialization |
| --- | --- | --- | --- | --- | --- | --- | --- | --- |
| Facebook |  |  |  |  |  |  |  |  |
| Twitter |  |  |  |  |  |  |  |  |
| YouTube |  |  |  |  |  |  |  |  |
| WhatsApp |  |  |  |  |  |  |  |  |
| Viber |  |  |  |  |  |  |  |  |
| Imo |  |  |  |  |  |  |  |  |
| Instagram |  |  |  |  |  |  |  |  |
| Tik Tok |  |  |  |  |  |  |  |  |
| Telegram |  |  |  |  |  |  |  |  |
| Snapchat |  |  |  |  |  |  |  |  |
| Others |  |  |  |  |  |  |  |  |

**Section III** - Usage of social media in health matters

1. Do you use social media to get information on the following health topics?

|  | Yes | No |
| --- | --- | --- |
| Overall health |  |  |
| First Aid/Emergency Treatment |  |  |
| Medicines for common ailments |  |  |
| Fat/Thin/Exercise |  |  |
| Healthy eating/nutrition counseling |  |  |
| Cancers |  |  |
| Related to Covid-19 |  |  |
| Men's Health/Women's Health |  |  |
| Childcare/health issues related to children |  |  |
| Reproductive Education |  |  |
| Appearance/face |  |  |

**Section IV**- Usage of social media in oral health matters

1. What applications do you use to obtain information on oral health.

|  | Yes | No |
| --- | --- | --- |
| Facebook |  |  |
| Twitter |  |  |
| YouTube |  |  |
| WhatsApp |  |  |
| Viber |  |  |
| Imo |  |  |
| Instagram |  |  |
| Tik Tok |  |  |
| Telegram |  |  |
| Snapchat |  |  |
| Others |  |  |

1. How often do you search/view oral health information?

| Not at all |  |
| --- | --- |
| Rarely |  |
| Every few months |  |
| Every few weeks |  |
| Every few days |  |

1. How do you feel about using social media platforms to get information about oral health?

…………………………………………………………………………………………………………………………………………………………………………………………………………………………………………………………………………………………………………………………………………………………………………………………………………………………………………………………………………

1. Have you had any experiences (positive or negative) while using social media related to oral health promotion? Write briefly.

…………………………………………………………………………………………………………………………………………………………………………………………………………………………………………………………………………………………………………………………………………………………………………………………………………………………………………………………………………

1. Do you find oral health information found on social media to be reliable or acceptable? 1–5 scale of agreement with oral health-related information; 1 strongly disagree and 5 strongly agree

| 1 | 2 | 3 | 4 | 5 |
| --- | --- | --- | --- | --- |
|  |  |  |  |  |

1. What are the advantages of using social media to obtain oral health information?

| Easy to access information |  |
| --- | --- |
| Fast and cost effective |  |
| Suitable for all ages |  |
| There is enough information |  |
| Clinics are not everywhere |  |
| Sharing experiences with friends is easy |  |

1. What kind of oral health information are you looking for?

| Oral Cancer |  |
| --- | --- |
| Oral hygiene (brushing, flossing, techniques etc.) |  |
| Oral health emergencies |  |
| Toothache Remedy |  |
| Tooth decay or gum disease |  |
| Oral health issues related to children |  |
| Finding the right dentist |  |
| Oral surgery |  |
| Risk habits (smoking, tobacco use, betel quid chewing, AN chewing) |  |
| Other reasons |  |

1. What are the disadvantages of using social media to obtain oral health information?

| Accuracy is questionable |  |
| --- | --- |
| Direct communication with experts is difficult |  |
| Not enough time to use social media |  |
| Not interested in oral health issues |  |
| The information available is insufficient |  |

| Accuracy/Transparency |  |
| --- | --- |
| Connection of person / expertise |  |
| Quality of information |  |
| Number of Followers/Subscribers/Likes (Popularity) |  |
| Referral of source by third party |  |
| Novelty / Latest |  |

1. What factors do you consider when evaluating the credibility of oral health information you find on social media?
2. What is your preference for receiving oral health information through social media? 1-5 scale; 1 is strongly not preferred at all, 5 is strongly preferred.

| 1 | 2 | 3 | 4 | 5 |
| --- | --- | --- | --- | --- |
|  |  |  |  |  |

1. What is your preference for website or social media platforms to disseminate oral health information?

| Website is preferred |  |
| --- | --- |
| Social media is a priority |  |
| Both website and social media are preferred |  |
| No one likes it |  |

| Yes |  |
| --- | --- |
| No |  |

1. Have you changed at least some of your oral health behaviors since accessing social media?
2. What is your preferred method of disseminating oral health information? (1-5 scale; 1 is strongly not preferred, 5 is strongly preferred)

|  | 1 | 2 | 3 | 4 | 5 |
| --- | --- | --- | --- | --- | --- |
| Short videos from experts |  |  |  |  |  |
| Dramas |  |  |  |  |  |
| Lectures |  |  |  |  |  |
| Slides |  |  |  |  |  |
| Short reading materials |  |  |  |  |  |
